# Shifting the focus: measuring positive childhood experiences and flourishing for holistic mental health in low and middle-income countries

**DOI:** 10.3389/fped.2025.1578485

**Published:** 2025-04-29

**Authors:** Miguel Landa-Blanco

**Affiliations:** School of Psychological Sciences, National Autonomous University of Honduras, Tegucigalpa, Honduras

**Keywords:** children, wellbeing, benevolent childhood experiences, flourishing, positive psychology

## 1 Introduction

Global child mental health research has long focused on the prevention and treatment of adverse childhood experiences (ACEs) and their associated risks (1). This focus is particularly salient in low- and middle-income countries (LMIC), given the high prevalence of structural and contextual adversities, such as poverty, conflict, and limited access to mental health services (2–4). While the study of ACEs has undeniably advanced our understanding of how early adversity shapes developmental trajectories (5), a narrow emphasis on risk factors leaves a critical gap in our knowledge: the role of positive experiences in fostering resilience and promoting long-term well-being.

In recent years, research has pointed to the protective value of benevolent childhood experiences (BCEs) —moments of safety, connection, and joy that act as counterbalances to adversity (6)—and flourishing, a multidimensional construct that reflects optimal functioning across emotional, social, and psychological domains (7). This article argues that incorporating these positive variables into research and intervention frameworks in LMIC is a scientific imperative and a strategic approach to promoting population-level resilience and well-being.

## 2 Rethinking mental health research in LMIC

In high-income contexts, the study of protective factors and positive outcomes has gained momentum (8, 9). However, in LMIC, mental health research has mainly remained deficit-focused—centered on reducing risk and alleviating psychopathology, with fewer studies focusing on protective factors (10). This focus is understandable given the complex and often severe challenges facing children in these settings, from chronic poverty and exposure to violence to fragmented health and education systems. Nevertheless, this risk-centric model provides only a partial view of mental health. Mental health is not merely the absence of illness; it is the presence of well-being (11). By fixating on pathology, interventions in LMIC risk neglecting the inherent strengths and adaptive capacities of individuals and communities, perpetuating a narrow understanding of mental health.

Flourishing represents a holistic state of well-being that includes emotional, psychological, and social dimensions. Unlike traditional measures of mental health, which focus on symptom reduction, flourishing emphasizes positive functioning—such as the ability to form meaningful relationships, experience life satisfaction, and engage productively in society (12–14). BCEs are relational and environmental experiences that create a sense of security, stability, and connection during childhood. Examples include having a supportive caregiver, feeling safe at home, participating in community activities, and experiencing unconditional love (6, 15). While adverse childhood experiences are potent predictors of negative outcomes (16, 17), BCEs have been shown to buffer their effects (18, 19), promoting resilience and humanism even in highly adverse environments (20).

In LMIC, BCEs often arise from informal support networks, such as extended family (21–23), religious communities (24), and local cultural practices that emphasize collective well-being. Measuring these positive experiences can illuminate critical pathways for intervention that do not rely solely on clinical or state-based services, which are often scarce in LMIC. Well-being in childhood is deeply embedded in family structures, community expectations, and cultural traditions, leading to significant variations in a nurturing and supportive environment. For instance, while some societies emphasize individualism, independence, self-expression, and personal achievement, others prioritize family cohesion, respect for elders, and collective responsibility (25, 26).

Considering cultural-specific practices may serve as unrecognized protective factors in LMIC but are rarely quantified in Western-centric measures, culturally adaptive research is essential to avoid imposing external definitions of well-being and ensure interventions resonate with community values. Participatory methods, such as co-designing measures with local stakeholders, could uncover context-specific strengths while maintaining scientific rigor. Overlooking these cultural dimensions risks imposing a universal framework that may not align with the lived realities of children from different backgrounds.

## 3 Policy and practice implications

Integrating BCEs and flourishing into child mental health frameworks in low- and middle-income countries requires a paradigm shift in both research and intervention. While the long-standing focus on adverse childhood experiences has been essential for identifying risk factors, it offers only a partial view of developmental trajectories. Effective policy and practice must move beyond mitigating harm to actively promoting resilience and well-being. This shift demands a multisectoral approach that prioritizes culturally grounded metrics, strengthens community-based assets, and aligns with global development goals to build resilience-enhancing ecosystems.

### 3.1 Building culturally valid metrics and research infrastructure

The absence of culturally validated metrics for BCEs and flourishing represents a critical gap in child mental health research. The Benevolent Childhood Experiences Scale (6), a ten-item questionnaire, provides a sensible and valuable starting point for assessing positive early experiences. However, as cultural contexts profoundly shape childhood development, well-being, and developmental trajectories, existing tools—primarily developed in high-income settings—may overlook protective factors rooted in diverse cultural practices. To address this, the BCEs Scale could serve as a core module, with additional items capturing culturally specific sources of resilience and support in LMIC. Similarly, researchers and policymakers can develop more inclusive and contextually relevant measures that reflect diverse developmental pathways by integrating culturally centered values into the conceptualization of flourishing (27). This approach strengthens the validity of flourishing as a construct and ensures that child-focused policies and interventions are more responsive to the unique needs and aspirations of different cultural groups.

Participatory research is crucial for co-creating context-specific indicators of well-being. Communal rituals, intergenerational activities, and shared family spaces may provide security and continuity amid adversity (28–30). Measuring flourishing at scale could also help policymakers identify successful interventions that promote positive outcomes, rather than focusing exclusively on reducing negative ones. Moreover, it aligns with the Sustainable Development Goals (SDG 3) by emphasizing health promotion and prevention.

Global agencies, such as the World Health Organization (WHO) and the United Nations International Children's Emergency Fund (UNICEF), must prioritize investments in developing culturally adapted measures. Ministries of health and education should embed these tools into national surveys to inform evidence-based resource allocation and policymaking. Such metrics would allow governments to identify not only areas of risk but also pockets of resilience, ensuring that interventions are both targeted and strengths-based. Clinicians, educators, and community health workers must integrate these tools into routine practice, enabling a comprehensive assessment of risks and resources (31, 32). For example, a child with limited parental support but strong ties to extended family and community networks might benefit from interventions that reinforce those existing supports rather than focusing solely on individual deficits. This shift from risk reduction to resilience promotion can transform how services are delivered and evaluated.

### 3.2 Leveraging local assets to build resilient communities

In LMIC, parenting and family interventions promoting children's well-being have shown promising results (33). Additionally, informal support systems—such as extended families and faith-based practices—often serve as a relevant source of care and protection for children (34, 35). Policies should formally recognize these community assets as integral components of the mental health infrastructure. Subsidizing and scaling community-led initiatives that align with local caregiving practices could significantly enhance population-level well-being (31). Effective practice requires moving beyond generic, imported models toward interventions that leverage these local strengths. Collaborating with community leaders to integrate traditional practices and resources into psychosocial support can increase acceptability and sustainability. This approach ensures that interventions resonate with local values and are more likely to succeed.

Embedding BCEs and flourishing within national strategies offers a pathway to advance the Sustainable Development Goals, particularly SDG 3 (health and well-being) and SDG 4 (quality education). Mental health promotion should not be siloed within health ministries but instead integrated across sectors. Education policies could incorporate social-emotional learning into school curricula to foster emotional resilience and peer support, while poverty-reduction programs could be paired with caregiver support initiatives to enhance family stability (36).

Multisectoral collaboration is critical (37). Ministries of health, education, housing, and social protection must work together to address the structural drivers of adversity while promoting positive outcomes. Schools, for instance, can become hubs for well-being by embedding strengths-based practices in daily routines. Teachers might encourage peer collaboration and foster classroom environments that promote creativity and problem-solving, while health workers use BCE data to connect families with community resources. Such systemic approaches ensure that flourishing becomes a measurable outcome, not an afterthought.

### 3.3 Shifting public narratives and expanding capacities

Public discourse around mental health in LMIC must evolve to emphasize resilience, connection, and community-driven well-being. National governments and NGOs should lead public awareness campaigns that highlight small but significant moments of joy, safety, and connection, shifting the focus from trauma to strength. Youth-led initiatives—such as art programs in post-conflict regions (38)—can be powerful examples of how communities generate healing and growth from within.

Equally critical is capacity building for frontline workers. Teachers, health professionals, and community leaders must be equipped with practical strategies to nurture BCEs and foster children's flourishing. Training programs should emphasize actionable skills, such as fostering caregiver-child bonding, promoting peer support, and leveraging play for emotional growth. This reorientation toward strengths-based care is scientifically justified and essential for achieving equitable outcomes in resource-constrained settings.

## 4 Discussion

Global child mental health research has predominantly emphasized adverse childhood experiences and their detrimental effects, particularly in low- and middle-income countries, where structural adversities are prevalent. While this focus has advanced understanding of risk factors, it neglects the role of positive experiences in fostering resilience and well-being. Recent research highlights the protective value of benevolent childhood experiences and flourishing, yet these constructs remain underutilized in LMIC research and policy frameworks.

This article argued for a paradigm shift integrating BCEs and flourishing into mental health assessments and interventions in LMIC. Flourishing encompasses emotional, social, and psychological dimensions of well-being, while BCEs—such as secure caregiving, community support, and cultural traditions—act as buffers against adversity. A deficit-focused approach limits intervention potential, whereas measuring these positive factors can inform strategies that build resilience through local strengths rather than solely addressing pathology. A critical challenge is the lack of culturally valid metrics for BCEs and flourishing in LMIC (39). Current measures, often developed in high-income contexts, may overlook community-specific protective factors. Contextually grounded research and participatory methodologies can enhance the validity and applicability of these constructs. Multisectoral policies that leverage community resources, integrate well-being metrics into national surveys, and align with global development goals can create sustainable resilience-enhancing ecosystems. Shifting the focus from risk reduction to strengths-based approaches in LMIC is a scientific and ethical imperative. By embracing a holistic framework, mental health interventions can move beyond mitigating harm to actively promoting thriving childhoods and lifelong well-being.

This paradigm shift honors LMIC communities as architects of their resilience, moving beyond imported models to solutions grounded in local realities. By centering resilience, connection, and collective agency, we can transform child mental health from a narrative of vulnerability to strength and possibility. The path forward is not only a matter of scientific progress but a necessary step toward advancing global health equity and ensuring that every child, regardless of circumstance, has the opportunity to thrive.
